# The anatomy of abscission zones is diverse among grass species

**DOI:** 10.1002/ajb2.1454

**Published:** 2020-03-23

**Authors:** Yunqing Yu, Patricia Leyva, Rachel L. Tavares, Elizabeth A. Kellogg

**Affiliations:** ^1^ Donald Danforth Plant Science Center 975 North Warson Road St. Louis MO 62132 USA; ^2^ California State University Long Beach 1250 Bellflower Blvd Long Beach CA 90840 USA; ^3^ Present address: University of Massachusetts Amherst Amherst MA 01003 USA

**Keywords:** abscission zone, grasses, lignin, morphological diversity, Poaceae, secondary cell wall, seed shattering, trait evolution

## Abstract

**Premise:**

Abscission zones (AZ) are specialized cell layers that separate plant parts at the organ junction upon developmental or environmental signals. Fruit or seed abscission has been well studied in model species because of its crucial role for seed dispersal. Previous work showed that AZ localization differs among species of Poaceae and that AZ formation is histologically and genetically distinct in three distantly related grass species, refuting the idea of a broadly conserved module. However, whether AZ structure is consistent within subfamilies is unknown.

**Methods:**

Eleven species were selected from six subfamilies of Poaceae, and their AZ was investigated using paraffin‐embedded, stained material. Observations were added from the literature for an additional six species. Data were recorded on AZ location and whether cells in the AZ were distinguishable by size or lignification. Characteristics of the AZ were mapped on the phylogeny using maximum likelihood.

**Results:**

Abscission zone anatomy and histology vary among species, and characteristics of the AZ do not correlate with phylogeny. Twelve of the seventeen studied species have an AZ in which the cells are significantly smaller than surrounding cells. Of these, eight have differential lignification. Differential lignification is often associated with differential cell size, but not vice versa.

**Conclusions:**

Neither smaller cells in the AZ nor differential lignification between the AZ and surrounding cells is required for abscission, although differential cell size and lignification are often correlated. Abscission zone anatomy does not correlate with phylogeny, suggesting its rapid change over evolutionary time.

Plants shed their unwanted organs such as senescent or infected leaves and floral parts, ripe fruits, and seeds in a highly controlled and programmed process known as abscission. Abscission occurs at the junction of two different organs in one to several specialized cell layers called the abscission zone (AZ). The AZ differentiates during organogenesis long before abscission. Upon developmental and/or environmental stimuli, AZ cells secrete cell‐wall‐hydrolyzing enzymes, resulting in degradation of cell wall components such as pectin and cellulose, and ultimately separation between cells (Patterson, 2001). In addition, disintegrated cells in the AZ may serve as a weak point for organ detachment, although the role of programmed cell death in abscission is still controversial (Sexton and Roberts, 1982). In tomato (*Solanum lycopersicum*) leaf and flower abscission, programmed cell death in the distal tissues of the AZ is required for abscission (Bar‐Dror et al., 2011). However, a study of the AZ of wild oat (*Avena fatua*) shows that the cells and nuclei remain intact even after abscission (Yeung et al., 1987). It is possible that there are different mechanisms for abscission in different species.

The cells that make up the AZ often show distinct anatomy from the surrounding cells, including small cell size, dense cytoplasm, lack of secondary cell wall, and highly branched plasmodesmata (Valdovinos and Jensen, 1968; Sexton and Roberts, 1982). The small cell size is thought to facilitate physical fracture of tissues in a linear path as compared to interlocked, large, elongated cells (Sexton and Roberts, 1982). In some species, loss of small cells in the AZ results in loss of abscission. For instance, the *blade‐on‐petiole 1* (*bop1*) *bop2* double mutants of *Arabidopsis thaliana* lack the small and cytoplasmically dense cells in the petal and sepal AZ and also lose abscission in these organs (McKim et al., 2008). Similarly, a knockout mutation in the *SHATTERING ABORTION1* gene (*SHAT1*) in rice (*Oryza sativa*) transforms the small AZ cells into elongated cells similar to the surrounding ones and completely disables grain abscission (Zhou et al., 2012), suggesting that the small cell size is a prerequisite for abscission in these species.

Cell wall composition may also play a crucial role in abscission. The AZ of some species is nonlignified but is surrounded by thickly lignified cells. Thin and nonlignified walls are more accessible for degradation, and differential lignification creates physical tension between cell types when tissue dries and loses turgor pressure, which eventually leads to breakage at the weakly attached AZ (Sexton and Roberts, 1982). Rice mutants with ectopic lignification in the AZ exhibit increased tensile strength of tissues holding the grain onto the plant and reduced or loss of grain abscission (Zhou et al., 2012; Yoon et al., 2014, 2017; Lv et al., 2018). In fruit abscission (also called dehiscence) of *Arabidopsis*, mutants with ectopic lignification in the AZ (Rajani and Sundaresan, 2001) or nonlignification in the cells adjacent to the AZ (Liljegren et al., 2000, 2004; Mitsuda and Ohme‐Takagi, 2008) both inhibit dehiscence, demonstrating the importance of differential lignification in the abscission process.

Despite the knowledge gained from studies of AZ development of model species (Ballester and Ferrándiz, 2017; Tranbarger et al., 2017), AZs may be diverse in morphology and developmental regulation (Doust et al., 2014; Yu and Kellogg, 2018; Yu et al., 2020). One example appears in Poaceae (the grass family), which consists of over 12,000 species including all cereal crops such as rice, wheat, and maize, and biofuel crops such as switchgrass (Kellogg, 2015). Grain abscission, also called shattering, is an undesirable trait in agriculture, and thus nonshattering cereals have been bred during thousands of years of crop domestication (Li and Olsen, 2016). Poaceae species have compound inflorescences, which consist of different orders of branches that bear the flower structures called spikelets. A spikelet is composed of a pair of bracts called glumes subtending one to multiple florets, which are the basic floral unit including the reproductive organs and a pair of floral bracts (lemma and palea) (Fig. 1).

**Figure 1:**
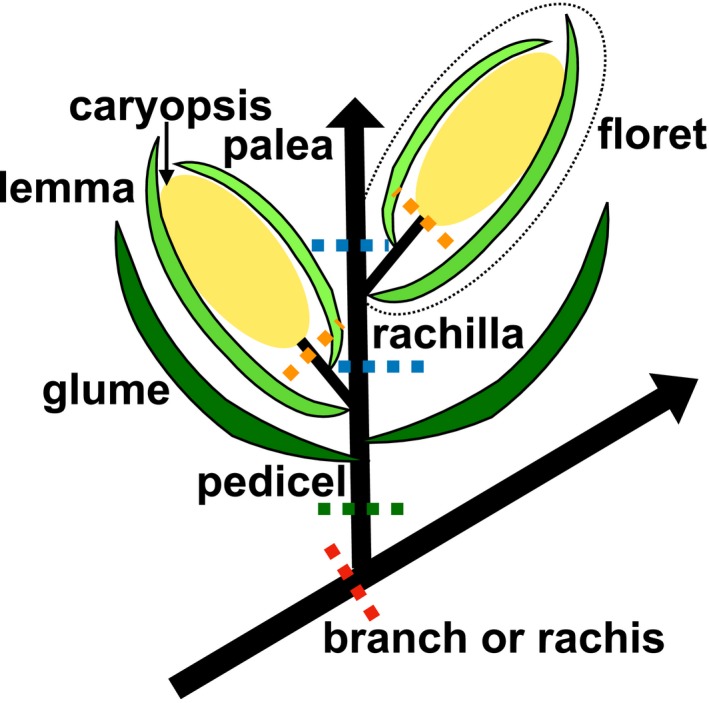
Spikelet structure. A spikelet consists of a pair of glumes subtending one or multiple florets. A floret (circled with dotted oval shape) consists of a pair of floral bracts called the lemma and palea, and reproductive organs above, which eventually develop into mature fruit (caryopsis). Depending on the species, the abscission zone (AZ) may be located in the branch or rachis (red dotted line), in the pedicel (green dotted line), in the rachilla (blue dotted lines), or above the lemma and palea (orange dotted lines).

The location of the AZ varies in different grass species, and its location is often used as a taxonomic character for identification of genera or tribes, described as disarticulation above or below the glumes (Barkworth et al., 2003, 2007). The AZ may be localized beneath a cluster of spikelets in the branches or rachis, beneath (proximal to) the spikelet in the pedicel, beneath the floret in the rachilla, or above the lemma and palea but below the fruit (caryopsis) (Fig. 1) (Doust et al., 2014; Yu et al., 2020). Previous work showed that the ancestral state is placement of the AZ in the rachilla (above the glumes) (Yu et al., 2020). Using three distantly related grass species including weedy rice (*Oryza sativa*) (Thurber et al., 2010), *Brachypodium distachyon*, and *Setaria viridis*, we demonstrated that the anatomy (Appendix S1) and gene network regulating the AZ differ among the three species (Yu et al., 2020). Although rice and *Brachypodium* share the same AZ position in the rachilla (Appendix S2) and small cells, they differ in cell wall composition (Appendix S1A–H). The rice AZ consists of a layer of nonlignified cells sandwiched between lignified cells (Appendix S1C), while the AZ of *Brachypodium* is the opposite, i.e., a layer of lignified cells is surrounded by less‐ or nonlignified cells (Appendix S1G). Different from the AZ of both rice and *Brachypodium*, the AZ of *Setaria viridis* is located in the pedicel (below the glumes), and its histology is not clearly distinct (Appendix S1, I–L) (Hodge and Kellogg, 2016; Yu et al., 2020). Studies of other species including wild barley (*Hordeum vulgare* subsp. *spontaneum*) (Pourkheirandish et al., 2015), *Elymus sibiricus* (Zhao et al., 2017), and *Lolium perenne* (Fu et al., 2018) further support the conclusion of divergence among different grass species, raising questions of how AZ development has evolved, whether it is possible to generalize from one species to other closely related species, and whether there is a phylogenetic trend in AZ anatomy across the grass family.

In this study, we enrich the histological data of the AZ across the Poaceae family and combine published data with our own new observations to analyze AZ anatomical patterns. We specifically focused on cell size and cell wall composition, two important factors that contribute to AZ function. To our surprise, we found no phylogenetic pattern. Closely related species have different AZ anatomy, and similar AZ anatomy may come from distantly related species, suggesting that AZ anatomy is highly subject to change and evolves rapidly through time.

## MATERIALS AND METHODS

### Plant materials and growth conditions

Material from 11 taxa was collected in this study. Taxa were chosen based on their phylogenetic position, and their availability for growing or collecting (Table 1). Inflorescences of *Bouteloua curtipendula*,* Chasmanthium latifolium*, and *Sporobolus heterolepis* were collected from the reconstructed prairie surrounding the Donald Danforth Plant Science Center (St. Louis, MO, USA). Voucher specimens of these three species were deposited in the Missouri Botanical Garden herbarium (St. Louis, MO, USA). Inflorescences of *Pharus latifolius* and *Lithachne pauciflora* were collected in the Climatron at the Missouri Botanical Garden. Other species were grown in greenhouses or growth chambers at the Donald Danforth Plant Science Center. *Streptochaeta angustifolia* (originally donated by Lynn Clark at Iowa State University) and *Andropogon gerardi* (big bluestem) were grown in a greenhouse with 30–40% humidity and 14 h light/10 h dark, with temperatures of 26°C day/20°C night. *Zea mays* subsp. *parviglumis* (teosinte, PI 384071) was grown in a growth chamber with 40–60% humidity and 12 h light/12 h dark, with temperatures of 31°C day/22°C night. *Eragrostis tef* (PI 524434) was grown in a greenhouse with 40–50% humidity and 14 h light/10 h dark, with temperatures of 28°C day/22°C night. *Triticum aestivum* subsp*. macha* (PI 611470) and *Avena murphyi* (PI 657363) were grown in a greenhouse with 40% humidity and 16 h light/8 h dark, with temperatures of 22°C day/20°C night.

**Table 1:** List of species used in this study.

Species	Subfamily	Tribe	Reference
*Streptochaeta angustifolia* Soderstr.	Anomochlooideae	Streptochaeteae	This study
*Pharus latifolius* L.	Pharoideae	Phareae	This study
*Andropogon gerardi* Vitman	Panicoideae	Andropogoneae	This study
*Zea mays *subsp. *parviglumis* Iltis & Doebley	Panicoideae	Andropogoneae	This study
*Setaria viridis* (L.) P.Beauv.	Panicoideae	Paniceae	Hodge and Kellogg, 2016
*Chasmanthium latifolium* (Michx.) H.O.Yates	Panicoideae	Chasmanthieae	This study
*Sporobolus heterolepis* (A.Gray) A.Gray	Chloridoideae	Zoysieae	This study
*Bouteloua curtipendula* (Michx.) Torr.	Chloridoideae	Cynodonteae	This study
*Eragrostis tef* (Zuccagni) Trotter	Chloridoideae	Eragrostideae	This study
*Oryza sativa* L.	Oryzoideae	Oryzeae	Yu et al., 2020
*Lithachne pauciflora* (Sw.) P.Beauv.	Bambusoideae	Olyreae	This study
*Brachypodium distachyon* (L.) P.Beauv.	Pooideae	Brachypodieae	Yu et al., 2020
*Triticum aestivum* subsp*. macha* Mackey	Pooideae	Triticeae	This study
*Hordeum vulgare* subsp. *spontaneum* (K.Koch) Asch. & Graebn.	Pooideae	Triticeae	Pourkheirandish et al., 2015
*Elymus sibiricus* L.	Pooideae	Triticeae	Zhao et al., 2017
*Lolium perenne* L.	Pooideae	Poeae	Fu et al., 2018
*Avena murphyi* Ladiz.	Pooideae	Poeae	This study

### Histology

Standard procedures were used for histological experiments (Ruzin, 1999). Specifically, multiple inflorescences were collected at various stages including heading, anthesis, and post anthesis. Anthesis (in *Avena murphyi* and *Sporobolus heterolepis)* and post‐anthesis (in all other species) stages with secondary cell wall differentiation were further pursued in this study. Inflorescence branches were fixed in FAA (50% ethanol, 5% glacial acetic acid, 10% formalin, and 35% DI water by volume) for a minimum of 24 h. The samples were then dehydrated with ethanol (50%, 70%, 85%, 95%, and three changes of 100% ethanol for a minimum of 3 h each), followed by an ethanol–Histoclear (National Diagnostics, Atlanta, GA, USA) series at 3:1, 1:1, 1:3 ratio and three changes of Histoclear for at least 3 h each. The Histoclear solution was then replaced by paraffin for a total of six paraffin replacements at 60°C.

Samples were embedded in molten Paraplast and sectioned into 10 μm thick ribbons using a Microm HM 355S microtome (ThermoFisher, Waltham, MA, USA). Hard tissues were immersed in ultrapure water for 1–2 weeks at room temperature to soften the tissues before sectioning (Ruzin, 1999). Sections were mounted and stained with Johansen's safranin O and fast green according to the standard protocol, but with NeoClear (Sigma‐Aldrich, St. Louis, MO, USA) instead of xylene (Ruzin, 1999). Slides were then mounted with a coverslip using Permount solution diluted with toluene. Safranin O is a cationic dye that stains acidic secondary cell components including lignin, chitin, and suberin. In contrast, fast green is an anionic counterstain to safranin O and dyes primary cell wall components (Ruzin, 1999). Slides were viewed using a Leica DM 750 microscope and images were captured using a Leica ICC50 HD camera and Leica Acquire v2.0 software (Leica Microsystems, Wetzlar, Germany).

### Characters recorded

In each section for each species, we noted several characters. (1) Position of the AZ relative to other parts of the spikelet. The positions were recorded by separating the diaspore from the plant stalk as described by Yu et al. (2020). The positions include “in the rachilla” (coded as 1), “in the pedicel”, “in the rachis or branches” and “above palea/lemma” (the last three coded as 2). The last three states were all identified as derived in the comprehensive study of Yu et al. (2020), who surveyed 250 representatives of the entire grass family. They are combined into a single state here to make the character binary, as required for subsequent analyses. (2) Cell size, whether the cells in the AZ were smaller than the surrounding cells or not. The area of the cells in the AZ and 2–3 layers above or below the AZ were quantified using ImageJ. A total of 29–93 cells from 2–4 specimens per species per cell position (AZ, above the AZ or below the AZ) was measured. One‐way ANOVA in R (v3.5.2) was used to determine any significant differences between cells at different positions. Abscision zone cells significantly smaller than the adjacent cells were defined by comparing the AZ cell area with the cells above or below the AZ (Tukey's honestly significant difference [HSD] test, *p* < 0.01), and coded as 1; otherwise, the cell size was coded as 2. (3) Pattern of lignification, comparing lignification of the AZ itself to the cells above and below. Cells in the vascular tissues and epidermal layer were not considered in the comparison. Nonlignified cells appear green (G) from fast green staining, and lignified cells appear magenta (M) from safranin O staining.

### Phylogenetic analyses

The phylogenetic tree was derived from a grass phylogeny based on complete plastomes from 250 taxa (Saarela et al., 2018), using the drop.tip function in the phytools package (Revell, 2012) in R (v3.5.2). Seven taxa in this study were not included in the original tree, but we inferred that their phylogenetic position could be represented adequately by a closely related species or subspecies for analysis (Appendix S2). The ancestral states of cell size and cell wall composition were reconstructed using the ace function in the phytools package (Revell, 2012) in R (v3.5.2) with the all‐rates‐different (ARD) model and equal‐rates (ER) model and discrete data type. Binary traits correlation was analyzed using Pagel's model implemented in the phytools package with both the ARD and ER model (Pagel, 1994; Revell, 2012) in R (v3.5.2).

## RESULTS

### Description of AZ anatomy and histology by species

#### Anomochlooideae

##### *Streptochaeta angustifolia* (tribe Streptochaeteae)

**Figure 2:**
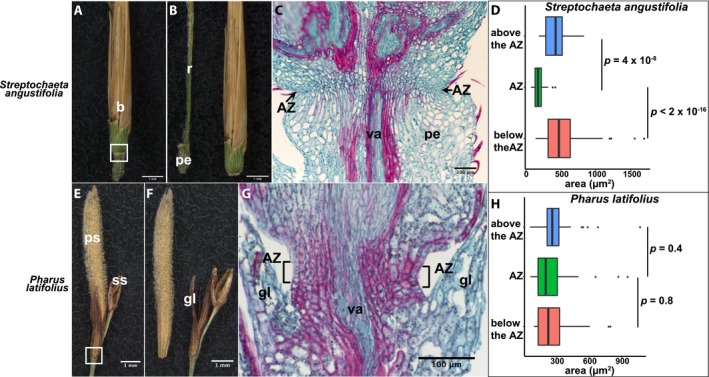
Abscission zone (AZ) of *Streptochaeta angustifolia* and *Pharus latifolius*. (A, B, E, F) Representative images of (A, E) attached and (B, F) detached (A, B) spikelet equivalent from the pedicel in *Streptochaeta angustifolia*, and (E, F) pisitillate floret from the rachilla in *Pharus latifolius*. Bars = 1 mm. (C, G) Safranin O and fast green staining of (C) *Streptochaeta angustifolia* and (G) *Pharus latifolius*, showing tissues in the white boxes in A and E, respectively. Bars = 100 μm. b, bract; gl, glume; pe, pedicel; ps, pistillate spikelet; r, rachis; ss, staminate spikelet; va, vascular bundle. (D, H) Quantification of the area of cells in the AZ and above and below the AZ in (D) *Streptochaeta angustifolia* and (H) *Pharus latifolius*. Tukey's HSD test was used to calculate *p* values.

Anomochlooideae is the subfamily sister to all other grass species and includes two genera, neither of which bears a typical grass spikelet. Species of *Streptochaeta* instead have a series of 11 or 12 bracts that surround a set of six stamens and three‐carpellary pistil, creating a structure sometimes called a spikelet equivalent (Judziewicz et al., 1999; Preston et al., 2009). The AZ of *Streptochaeta angustifolia* is located in the pedicel right below the outermost bracts (Fig. 2A, B; Appendix S2). The cells of the AZ are noticeably smaller and rounder than those above and below, and this pattern is more pronounced in the outside layers of cells than the cells close to the vascular tissues (Fig. 2C). Quantification of cell area shows that the cells in the AZ are significantly smaller than those above and below the AZ (Tukey's HSD test, *p* = 4 × 10^‐8^ and <2 × 10^‐16^, respectively) (Fig. 2D; Appendix S3). The outside layers of cells are not lignified in either the AZ or the surrounding cells as shown by fast green staining, while the cells close to the vascular tissues are lignified in the AZ and the cells above and below it, as shown by the magenta color from safranin O staining. Thus, little differential lignification occurs between the AZ and neighboring cells (Fig. 2C).

#### Pharoideae

##### *Pharus latifolius* (tribe Phareae)

*Pharus latifolius* has paired staminate and pistillate spikelets, but only the pistillate spikelets were analyzed (Fig. 2E, F). Each spikelet has a single floret with the AZ located below the floret and above the glumes (Fig. 2E, F; Appendix S2). No distinct cell layers were observed in the AZ, except that the region where abscission occurs forms a “neck” that is narrower than the tissues above and below it. We quantified the cell layers at the neck region as the AZ (square brackets in Fig. 2G) and the 2–3 layers of cells above and below the AZ. There is no significant difference in the cell size between the AZ and the cells above and below it (Tukey's HSD test, *p* = 0.4 and 0.8, respectively; Appendix S3). The cells in the AZ and surrounding the AZ are all lignified shown by the magenta color (Fig. 2G).

#### Panicoideae

##### *Andropogon gerardi* (tribe Andropogoneae)

**Figure 3:**
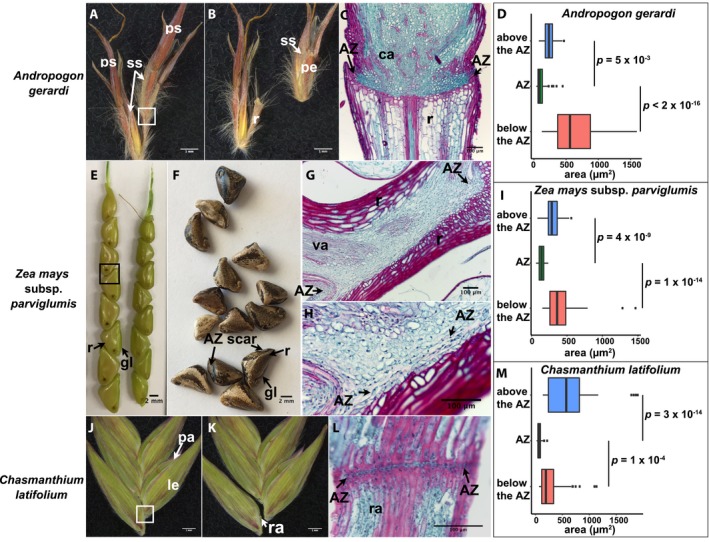
Abscission zone (AZ) of species in subfamily Panicoideae. (A, B, E, F, J, K) Representative images of (A, E, J) attached or (B, F, K) detached (A, B) spikelet pair of *Andropogon gerardi*, (E, F) spikelets of *Zea mays* subsp. *parviglumis*, and (J, K) florets of *Chasmanthium latifolium*. (A, B) Two spikelet pairs of *Andropogon gerardi*. The AZ is located in the rachis below the sessile spikelet. Bars = 1 mm. (E, F) Inflorescences of *Zea mays* subsp. *parviglumis*. The AZ is located in the rachis between the spikelets. Bars = 2 mm. (J, K) Several florets of *Chasmanthium latifolium*. The AZ is located in the rachilla between florets. Bars = 1 mm. (C, G, L) Safranin O and fast green staining of tissues in the white/black boxes in A, E, and J, respectively. (H) A more highly magnified image of panel G showing the flattened AZ cells. ca, callus; gl, glume; le, lemma; pa, palea; pe, pedicel; ps, pedicellate spikelet; r, rachis; ra, rachilla; ss, sessile spikelet. (D, I, M) Quantification of the area of cells in the AZ, and above and below the AZ in (D) *Andropogon gerardi*, (I) *Zea mays* subsp. *parviglumis*, and (M) *Chasmanthium latifolium*. Tukey's HSD test was used to calculate *p* values.

A bisexual sessile spikelet and one or two male pedicellate spikelets form the dispersal unit in this species, with the AZ located in the rachis below the spikelet pair (Fig. 3A, B; Appendix S2). The sessile spikelet was used for histological analysis. The junction between the rachis and the sessile spikelet is differentially lignified, with the cells in the rachis being highly lignified while those at the base of the spikelet (callus) are not (Fig. 3C). The cells in the lowermost AZ layers are highly significantly smaller than the elongated cells below (Tukey's HSD test, *p* < 2 × 10^‐16^), and are slightly but significantly smaller than the nonlignified and cytoplasmically dense cells in the callus above the AZ (Tukey's HSD test, *p* = 5 × 10^‐3^) (Fig. 3C, D; Appendix S3).

##### *Zea mays* subsp. *parviglumis* (tribe Andropogoneae)

In this species (commonly known as teosinte), only the sessile spikelet develops to maturity and is pistillate, while the other spikelet is aborted during early development (Sundberg and Orr, 1990). The AZ is located in the rachis just below the spikelet pair (Fig. 3E, F; Appendix S2). Similar to *Andropogon gerardi*, the AZ is at the junction of large and highly lignified cells in the rachis and small nonlignified cells on the other side (Fig. 3G). Closer observation shows that the cells at the AZ are thinner compared to the round cells above and are aligned longitudinally along the presumed breakage line (Fig. 3H). The thin AZ cells are significantly smaller than those above and below the AZ (Tukey's HSD test, *p* = 4 × 10^‐9^ and 1 × 10^‐14^, respectively) (Fig. 3I; Appendix S3).

##### *Chasmanthium latifolium* (tribe Chasmanthieae)

Our previous work has shown that the ancestral state of AZ position for Poaceae as a whole is likely located in the rachilla, while the core Panicoideae species have an AZ position in the pedicel or rachis, a derived state (Yu et al., 2020). *Chasmanthium latifolium* belongs to one of the lineages sister to the core and has the ancestral AZ position in the rachilla below each floret (Fig. 3J, K; Appendix S2) (Yu et al., 2020). The AZ is composed of one or two layers of small, nonlignified cells surrounded by large and lignified cells (Tukey's HSD test, *p* = 3 × 10^‐14^ and 1 × 10^‐4^ between the cell area of AZ and cells above and below it, respectively) (Fig. 3L, M; Appendix S3), similar to that of rice (Appendix S1, C, D).

#### Chloridoideae

##### *Sporobolus heterolepis* (tribe Zoysieae)

**Figure 4:**
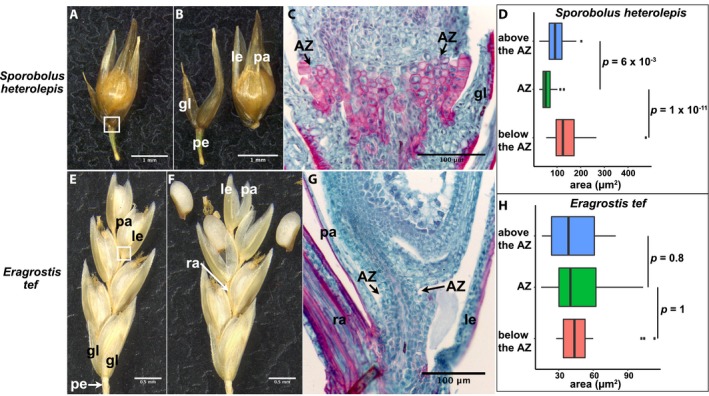
Abscission zone (AZ) of *Sporobolus heterolepis* and *Eragrostis tef* in subfamily Chloridoideae. (A, B, E, F) Representative images of (A, E) attached and (B, F) detached (A, B) floret of *Sporobolus heterolepis*, and (E, F) florets of *Eragrostis tef* from the rachilla. (A, B) Bars = 1 mm. (E, F) Bars = 0.5 mm. (C, G) Safranin O and fast green staining of tissues in the white boxes in images A and E, respectively. Bars = 100 μm. gl, glume; le, lemma; pa, palea; pe, pedicel; ra, rachilla. (Note that panel G shows a dark red rectangle in the lower left corner. It was in the original image and seems to be a bit of detached tissue. It is not the result of image manipulation.) (D, H) Quantification of the area of cells in the AZ and above and below the AZ in (D) *Sporobolus heterolepis*, and (H) *Eragrostis tef*. Tukey's HSD test was used to calculate *p* values.

The spikelet of this species has a single floret, with the AZ located above the glumes in the rachilla (Fig. 4A, B; Appendix S2). The lignified rachilla below the AZ is clearly distinct from the base of the floret above the AZ. The nonlignified AZ cells appear to be thinner than the surrounding cells and are significantly smaller than the cells above and below the AZ (Tukey's HSD test, *p* = 6 × 10^‐3^ and 1 × 10^‐11^, respectively) (Fig. 4C, D; Appendix S3).

##### *Eragrostis tef* (tribe Eragrostideae)

A spikelet of *Eragrostis tef* is composed of multiple florets subtended by a pair of glumes. Although the common ancestor of Tribe Eragrostideae and the closely related tribes is inferred to have had an AZ located in the rachilla, the AZ of *Eragrostis tef* is located above the lemma and palea below the caryopsis, which is a rare AZ position in the grass family (Fig. 4E, F; Appendix S2) (Yu et al., 2020). Histological analysis shows that disarticulation is likely to occur at the weak and narrow region below the fruit. The cells in this region are small and nonlignified, but not distinguishable from the cells above and below the AZ (Tukey's HSD test, *p* = 0.8 and 1, respectively) (Fig. 4G, H; Appendix S3).

##### *Bouteloua curtipendula* (tribe Cynodonteae)

**Figure 5:**
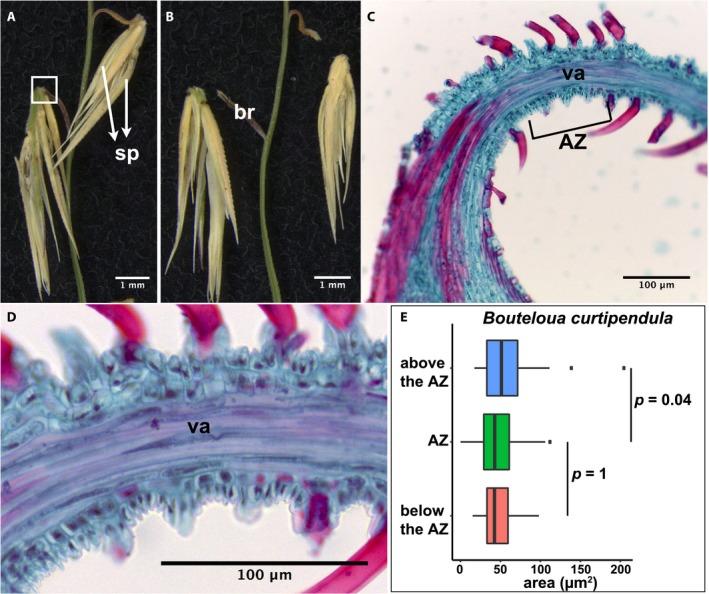
Abscission zone (AZ) of *Bouteloua curtipendula* in subfamily Chloridoideae. (A, B) Representative images of (A) attached and (B) detached spikelet clusters from the branch. Bars = 1 mm. (C) Safranin O and fast green staining of tissues in the white box in A. (D) A more highly magnified image of the AZ shown in C. (C, D) Bars = 100 μm. br, branch; sp, spikelet; va, vascular bundle. (E) Quantification of the area of cells in the AZ and above and below the AZ. Tukey's HSD test was used to calculate *p* values.

Although most Cynodonteae species have an AZ in the rachilla, the disarticulation of *Bouteloua curtipendula* occurs in the branch at the base of a cluster of spikelets (Fig. 5A, B; Appendix S2), a derived state (Yu et al., 2020). The branch that bears the spikelet clusters is thin and reflexed, and the breakage occurs at the bending point of the branch (Fig. 5A–C). We find no histological distinction in the region where abscission occurs (Fig. 5C–E). The nonvascular cells in that part of the branch are small, square‐shaped, and nonlignified (Fig. 5D). The cell sizes are comparable with the surrounding cells (Tukey's HSD test, *p* > 0.01) (Fig. 5E; Appendix S3).

#### Bambusoideae

##### *Lithachne pauciflora* (tribe Olyreae)

**Figure 6:**
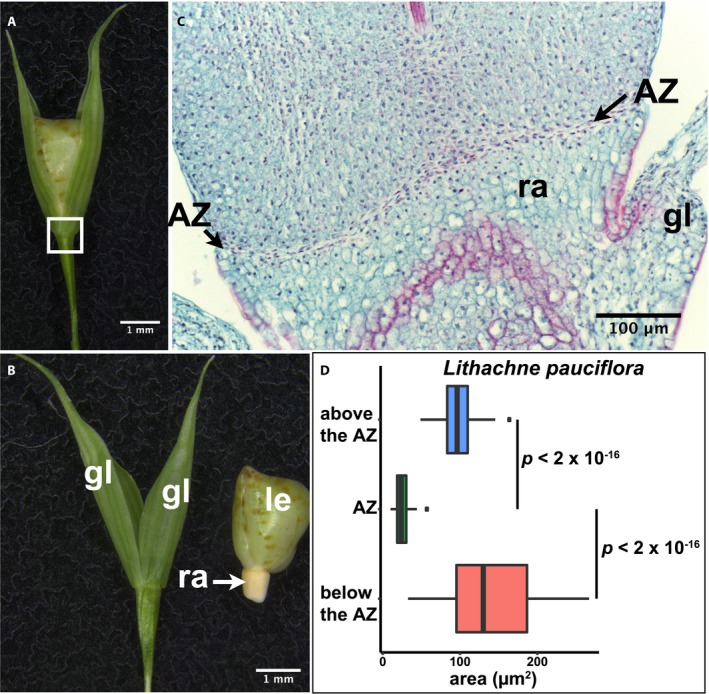
Abscission zone (AZ) of *Lithachne pauciflora* in subfamily Bambusoideae. (A) Representative image of a spikelet. (B) Floret detached from the base of rachilla. (A, B) Bars = 1 mm. (C) Safranin O and fast green staining showing tissues in the white box in A. Bar = 100 μm. gl, glume; le, lemma; ra, rachilla. (D) Quantification of the area of cells in the AZ and above and below the AZ. Tukey's HSD test was used to calculate *p* values.

*Lithachne pauciflora* has staminate and pistillate spikelets; here we examined the pistillate ones. The AZ is located above the glumes and below the single floret (Fig. 6A, B; Appendix S2). The AZ cells are noticeably smaller and more densely packed than the cells above and below (Tukey's HSD test, *p* < 2 × 10^‐16^ for both) (Fig. 6C, D; Appendix S3), but little lignification is observed in either the AZ or surrounding cells (Fig. 6C).

#### Pooideae

##### *Triticum aestivum* subsp*. macha* (tribe Triticeae)

**Figure 7:**
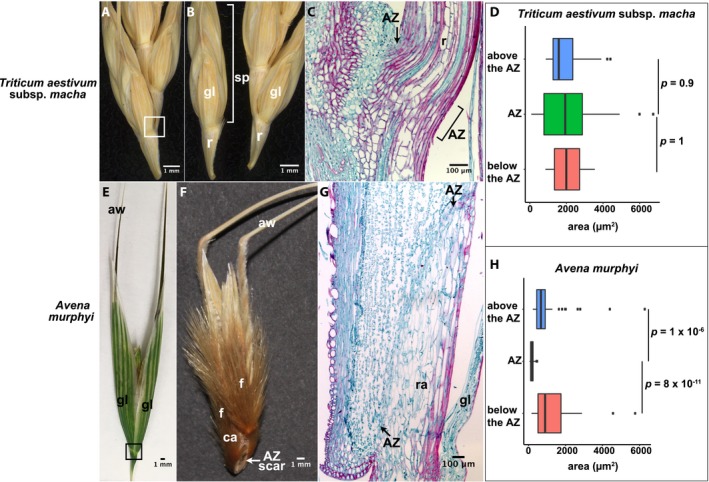
Abscission zone (AZ) of *Triticum aestivum* subsp*. macha and Avena murphyi* in subfamily Pooideae. (A, B, E, F) Representative images of (A, E) attached and (B, F) detached (A, B) spikelets of *Triticum aestivum* subsp. *macha* and (E, F) florets of *Avena murphyi*. (A, B, E, F) Bars = 1 mm. (C, G) Safranin O and fast green staining of tissues in the white/black boxes in A and E, respectively. Bars = 100 μm. aw, awn; ca, callus; f, floret; gl, glume; r, rachis; ra, rachilla; sp, spikelet. (D, H) Quantification of the area of cells in the AZ and above and below the AZ in (D) *Triticum aestivum* subsp. *macha* and (H) *Avena murphyi*. Tukey's HSD test was used to calculate *p* values.

*Triticum aestivum* subsp*. macha* has multiple florets in each spikelet, and the AZ is located in the rachis below the spikelet (Fig. 7A, B). The cells in the AZ are elongated similarly to the surrounding ones (Tukey's HSD test, *p* = 0.9 and 1 between cells in the AZ and those above and below it, respectively), and lignification is observed throughout the rachis including the AZ. Thus, the AZ of this species is not histologically distinct (Fig. 7C, D; Appendix S3).

##### *Avena murphyi* (tribe Poeae)

*Avena murphyi* often has two fertile florets, plus a few less‐developed sterile florets at the top. The AZ is located below the lowest floret in the rachilla and above the glumes (Fig. 7E, F; Appendix S2), where a thin layer of small cells is clearly distinguishable from the cells above and below (Tukey's HSD test, *p* = 1 × 10^‐6^ and 8 × 10^‐11^, respectively) (Fig. 7G, H; Appendix S3). However, no lignification is observed in the AZ or the surrounding cells except in the epidermis (Fig. 7G).

### Cell size and cell wall composition of the AZ vary over evolutionary time

**Figure 8:**
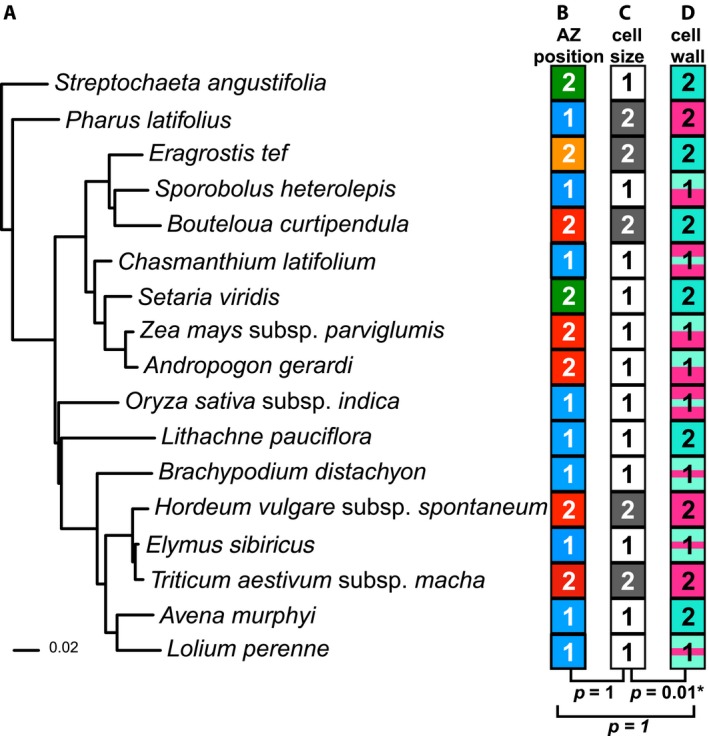
Abscission zone (AZ) characters mapped on a phylogenetic tree. (A) Phylogenetic tree pruned from Saarela et al. (2018). (B) AZ positions. Red box, in the rachis or branch; blue box, in the rachilla; orange box, above palea and lemma; green box, in the pedicel. 1, in the rachilla; 2, not in the rachilla (in the rachis, in the pedicel or above palea/lemma). (C) Cell size. White box labeled with 1, AZ cells smaller than the surrounding cells. Dark grey boxes labeled with 2, AZ cells the same size as the surrounding cells. (D) Cell wall composition. Coding colors match the safranin O and fast green staining colors. Green box, no lignin in AZ or surrounding cells (G); magenta box, lignin in both the AZ and the surrounding cells (M); green‐magenta box, nonlignification in the cells above the AZ and lignification in the cells below the AZ (GM); magenta‐green‐magenta box, nonlignified AZ cells sandwiched between lignified cells (MGM); green‐magenta‐green box, lignified AZ cells sandwiched between nonlignified cells (GMG). 1, Differential lignification including GM, GMG and MGM; 2, nondifferential lignification including G and M. *P* values were calculated from binary traits correlation test using Pagel's model with equal‐rates model option.

In a comprehensive study of the entire grass family, Yu et al. (2020) showed that the ancestral state of the AZ position is located in the rachilla below the floret. To further test for phylogenetic patterns in AZ anatomical traits, we categorized AZ anatomy based on cell size and cell wall composition. The cell size of the AZ was divided into two states: significantly smaller than the adjacent cells (Tukey's HSD test, *p* < 0.01) (Fig. 8C, labeled as 1), and about the same size as the adjacent cells (Tukey's HSD test, *p* > 0.01) (Fig. 8C, labeled as 2). While more than half of the species (12 of 17) have smaller AZ cells, both cell size states were observed in subfamilies Chloridoideae and Pooideae (Fig. 8A, C). In subfamily Chloridoideae, the AZ cells of *Sporobolus heterolepis* are smaller than the neighboring cells (Fig. 4C, D), while the AZ cells of *Eragrostis tef* (Fig. 4G, H) and *Bouteloua curtipendula* are not (Fig. 5C–E). In subfamily Pooideae, although *Elymus sibiricus* (Zhao et al., 2017), *Avena murphyi* (Fig. 7G, H), and *Lolium perenne* (Fu et al., 2018) have smaller AZ cells, *Hordeum vulgare* subsp. *spontaneum* (Pourkheirandish et al., 2015) and *Triticum aestivum* subsp. *macha* (Fig. 7C, D) do not show this pattern (Fig. 8A, C; Appendix S4). In contrast, we observed smaller AZ cells in all four species tested in subfamily Panicoideae (Figs. 3, 8A, 8C; Appendix S1, I–L, S4). We note that although the AZ of *Setaria viridis* is not obviously differentiated based on visual inspection (Hodge and Kellogg, 2016; Yu et al., 2020), quantification of cell size did show significant differences between the cells in the AZ and those below it (Tukey's HSD test, *p* = 9 × 10^‐5^) (Appendix S1, K, L). Overall, the ancestral reconstruction of AZ cell size is ambiguous, indicating approximately equal probabilities of the ancestor having had smaller or approximately equal cell sizes (Appendix S4).

Since different cell wall compositions in the AZ may indicate different biochemical and physical mechanisms for abscission, we classified cell wall composition into five states as follows (color coding matches stain colors in cell wall): neither the AZ or the surrounding cells are lignified (green, G), both the AZ and the surrounding cells are lignified (magenta, M), the upper cells are nonlignified and lower cells lignified (green‐magenta, GM), nonlignified AZ cells sandwiched between lignified cells (magenta‐green‐magenta, MGM), and lignified AZ cells sandwiched between nonlignified cells (green‐magenta‐green, GMG) (Fig. 8D; Appendix S5). The number of species in each category are 6 in G, 3 in M, 3 in GM, 2 in MGM, and 3 in GMG. Interestingly, the species most studied for AZ development, rice (Appendix S1C), only has one species (*Chasmanthium latifolium*) sharing the same lignification pattern (Fig. 3L), but the two taxa are unrelated (Fig. 8A, D). Again, we observed no phylogenetic patterns of cell wall composition. Species from the same tribes and subfamilies may exhibit different character states, and the same cell wall composition pattern may appear in distantly related species (Fig. 8A, D; Appendix S5), suggesting rapid change over evolutionary time and morphological convergence across subfamilies.

### Cell size and cell wall composition of AZ may be phylogenetically correlated

The shift of the AZ from its ancestral position in the rachilla (Yu et al., 2020) to other positions may accompany modifications in developmental programs, and thus the anatomical traits of the AZ. To test whether there is a correlation between AZ morphological traits in a phylogenetic context, we further simplified the traits into binary characters. The AZ position is categorized as in the rachilla (Fig. 8B, labeled as 1) vs. elsewhere (in the rachis or branches, in the pedicel or above the palea/lemma, Fig. 8B, labeled as 2). The cell wall composition is categorized as differential lignification (including GM, GMG, and MGM, Fig. 8D, labeled as 1), which suggests that abscission is facilitated by differential cell wall strength, and no differential lignification (including G and M, Fig. 8D, labeled as 2), which suggests that disarticulation occurs with a mechanism other than differential cell wall strength. We found no correlation between AZ position vs. cell size (*p* = 0.52 or 1, ARD or ER model, respectively), or AZ position vs. cell wall composition (*p* = 0.55 or 1, ARD or ER model, respectively) (Appendix S6). However, we found a significant correlation between AZ cell size vs. cell wall composition under the ER model (*p* = 0.01) but not under the ARD model (*p* = 0.08) (Appendix S6). Specifically, there are eight cases of a combination of differential cell size and differential lignification (Fig. 8C, D, coded as 1 1), five cases of same cell size and nondifferential lignification (Fig. 8C, D, coded as 2 2), and four cases of differential cell size but nondifferential lignification (Fig. 8C, D, coded as 1 2). However, we did not observe any species with the same cell size but differential lignification (Fig. 8C, D, coded as 2 1).

## DISCUSSION

### Cell size of the AZ varies among different grass species

According to the literature, a typical AZ is composed of small, nonlignified cells with dense cytoplasm as shown in model species including common bean (*Phaseolus vulgaris*) (Brown and Addicott, 1950), *Arabidopsis thaliana* (Patterson, 2001), and rice (Li and Olsen, 2016). Our recent study showed that these distinct anatomical characters are not required for abscission in all grass species (Yu and Kellogg, 2018; Yu et al., 2020). Here, we analyzed a total of 17 species across seven subfamilies in Poaceae and showed even more diversity in AZ anatomy than previously recognized.

Although most species presented here (12/17) have smaller cells in the AZ compared to the surrounding ones, we found no differentiation in cell size in Pharoideae (*Pharus latifolius*) (Fig. 2G, H), Chloridoideae (*Eragrostis tef* and *Bouteloua curtipendula*) (Figs. 4G, 4H, 5C–E) and Pooideae (*Hordeum vulgare* and *Triticum aestivum* subsp. *macha*) (Pourkheirandish et al., 2015) (Figs. 7C, 7D, 8A, 8C). We conclude that cell size differentiation is not necessary for a functional AZ and that changes in size differentiation occur repeatedly during evolution. It is worth noting that our categorization is based on relative cell size compared to the surrounding cells. Most species in which AZ cells are not distinguishable by size actually have small cells throughout the tissue, a pattern that is especially obvious in *Eragrostis tef* (Fig. 4G, H) and *Bouteloua curtipendula* (Fig. 5C–E). Therefore, although this pattern does not create an obvious weak cell layer for breakage, it does not violate the hypothesis that small cells facilitate breakage in a linear path (Sexton and Roberts, 1982).

### The cell wall composition of the AZ is diverse in different grass species

A pattern of nonlignified cells next to lignified cells is important for a functional AZ in fruits of *Arabidopsis thaliana* and rice (Liljegren et al., 2000, 2004; Rajani and Sundaresan, 2001; Mitsuda and Ohme‐Takagi, 2008; Zhou et al., 2012; Yoon et al., 2014, 2017). However, our study found approximately equal numbers of species with and without differential lignification (8 vs. 9, respectively) (Fig. 8A, D; Appendix S5), reinforcing the idea that differential lignification (including GM, GMG, and MGM) is not required for abscission, at least in some grass species.

It is important to note that safranin O is not specific to lignin, but also stains other secondary walls such as suberin. Also, there could be differences in other cell wall components that are not distinguished by the stains used here. Therefore, there may be additional diversity in cell wall composition that our methods would not detect, and lack of differentiation by safranin O and fast green staining does not preclude differences in some other respects.

### Cell size is likely to be correlated with lignification pattern in the AZ

Early observations proposed that cell growth is suppressed in the AZ, and thus, the AZ cells retain a morphology similar to that of meristems, without cell expansion or lignification (Sexton and Roberts, 1982). However, species including *Brachypodium distachyon* (Appendix S1G), *Elymus sibiricus* (Zhao et al., 2017), and *Lolium perenne* (Fu et al., 2018) exhibit a small cell size in the AZ, and yet are more lignified than the adjacent larger cells, suggesting that larger cells do not always correlate with lignification. Instead, differences in cell size correlate with differential lignification (Fig. 8). In particular, if AZ cells do not differ in size from surrounding tissues, they also fail to differ in lignification (G or M). Likewise, if AZ cells differ in lignification (GM, GMG, MGM), they also always differ in cell size (Fig. 8). However, if AZ cells differ in size, they may or may not differ in lignification. In other words, differential lignification coincides with differential cell expansion, but cell expansion may occur with or without lignification. Consistent with this observation, the reduced shattering rice mutants *sh4*,* sh5*, and *Obsh3* with ectopic lignification in the AZ retain the smaller cell size (Zhou et al., 2012; Yoon et al., 2014; Lv et al., 2018), while the *shat1* mutant loses the small cell size and also the differential lignification (MGM) pattern (Zhou et al., 2012). Since the secondary cell wall is deposited after cell expansion ceases, the diverse cell anatomy seen in different grass species may be related to the timing of primary cell expansion and secondary wall deposition.

### Different grasses may have evolved distinctive mechanisms for abscission

Abscission is a highly conserved process in plants and is a combination of biochemical processes that involve cell wall degradation and cell disintegration, and physical tension between tissues. However, whether plants share a common genetic pathway is still questionable. Previous work on the transcriptome of the AZ and surrounding tissues in rice, *Brachypodium*, and *Setaria* found sets of AZ‐specific genes in each. While the genes expressed in the AZ and its surrounding tissues are largely conserved, the genes whose expression specifically characterizes the AZ differ almost completely among the three, with only a handful (fewer than five) shared in all three species (Yu et al., 2020). Consistent with this result, the extensive morphological, anatomical, and histological diversity presented here rules out the possibility that different grass species utilize the same physical mechanism to disarticulate their fruits. In special cases, such as *Eragrostis tef* (Fig. 4E–H) and *Bouteloua curtipendula* (Fig. 5), both of which have AZ positions different from those of their close relatives, the AZ is not distinct in either cell size or cell wall composition. Since disarticulation occurs at the weakest point when plants are dried, it is possible that breakage is simply caused by a physical force without any enzymatic activity. If this is the case, the original AZ may have been lost during evolution since it was no longer under selection. Therefore, the evolution of the AZ may involve repeated loss of the ancestral AZ and creation of new AZ either at a new location or with a new breakage mechanism.

It is unclear why natural selection would lead to such diverse morphological structures and gene regulatory networks to control a highly conserved function. It is possible that anatomical patterns correlate with timing or ease of seed disarticulation. For example, we observed that species including *Brachypodium distachyon*,* Pharus latifolius*,* Chasmanthium latifolium*, and *Triticum aestivum* subsp*. macha* tend to shatter at late developmental stages when spikelets are fully mature and dried, and require greater force for diaspore separation than other species such as *Setaria viridis* and *Avena murphyi*. It is interesting that *Brachypodium distachyon*,* Pharus latifolius*, and *Triticum aestivum* subsp*. macha* also have lignified AZs, suggesting that lignification may play a role in the ease of shattering. However, the AZ of *Chasmanthium latifolium* is nonlignified, and yet it is late to shatter, indicating other factors are involved in controlling the process.

Another possibility is that only a few key enzymes or other signaling proteins are required for abscission and that they are masked by the large numbers of genes that control morphology. If the function of abscission does not rely on a particular morphological trait, then the morphology is more likely to diversify during evolution. Further studies on enzymatic activities and cell wall modification will further advance our knowledge of abscission mechanisms and AZ evolution.

## CONCLUSIONS

AZ anatomy and histology are highly diverse in the grass family, suggesting different mechanisms for abscission. Differential cell size and cell wall composition of the AZ are often correlated, but neither is required for abscission. AZ anatomy exhibits little phylogenetic pattern at the subfamily or tribe level, indicating rapid change of AZ anatomy over evolutionary time in Poaceae.

## AUTHOR CONTRIBUTIONS

E.A.K. designed the research and secured funding. E.A.K. and Y.Y. designed the experimental approach. Y.Y., P.L., and R.T. performed the experiments. Y.Y. performed the phylogenetic analysis. Y.Y., E.A.K., and P.L. wrote the manuscript. All authors contributed to ideas, discussed the results, and edited the manuscript.

## Supporting information


**APPENDIX S1.** Abscission zone (AZ) position and anatomy in *Oryza sativa*,* Brachypodium distachyon*, and *Setaria viridis* (reproduced with permission from Yu et al., 2020).

**APPENDIX S2.** Species used and their morphological characters.

**APPENDIX S3.** Values of one‐way ANOVA and Tukey's HSD (honestly significant difference) tests of cell area in, above, and below the AZ.

**APPENDIX S4.** Ancestral state reconstruction of AZ cell size.

**APPENDIX S5.** Ancestral state reconstruction of cell wall composition.

**APPENDIX S6.** Values of Pagel's binary character correlation test using both all‐rates‐different (ARD) model and the equal‐rates (ER) model.
