# Supplementary material for: The anatomy of abscission zones is diverse among grass species
Source: Am J Bot. 2020 Mar 23;107(4):549–61. doi: 10.1002/ajb2.1454 (PMC7217018; doi:10.1002/ajb2.1454)
Supplement: Supplementary file 4 — APPENDIX S4. Ancestral state reconstruction of AZ cell size. [file AJB2-107-549-s004.pdf]

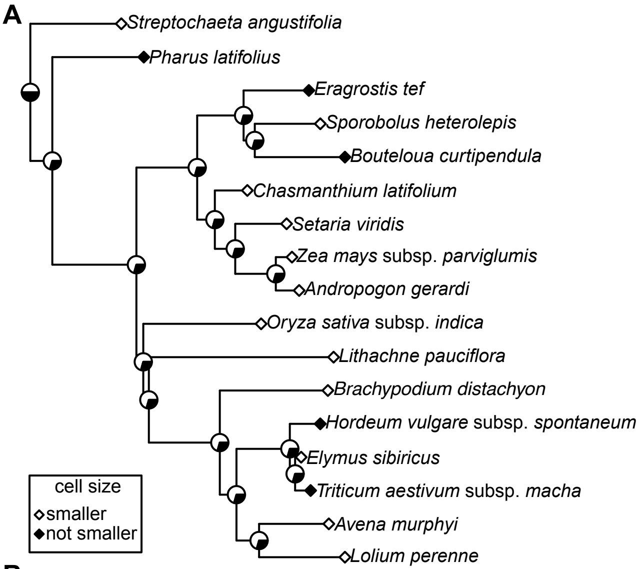

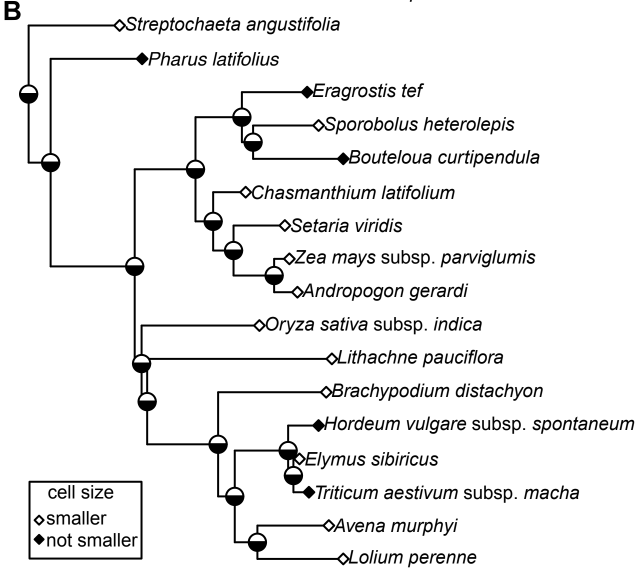

Appendix S4. Ancestral state reconstruction of AZ cell size. (A) All-rates-different model. (B) Equal-rates model. White color, AZ cells smaller than the surrounding cells (smaller, Tukey's HSD, $p < 0.01$). Black color, AZ cells the same size as the surrounding cells (not smaller, Tukey's HSD, $p > 0.01$).
