# Supplementary material for: The anatomy of abscission zones is diverse among grass species
Source: Am J Bot. 2020 Mar 23;107(4):549–61. doi: 10.1002/ajb2.1454 (PMC7217018; doi:10.1002/ajb2.1454)
Supplement: Supplementary file 5 — APPENDIX S5. Ancestral state reconstruction of cell wall composition. [file AJB2-107-549-s005.pdf]

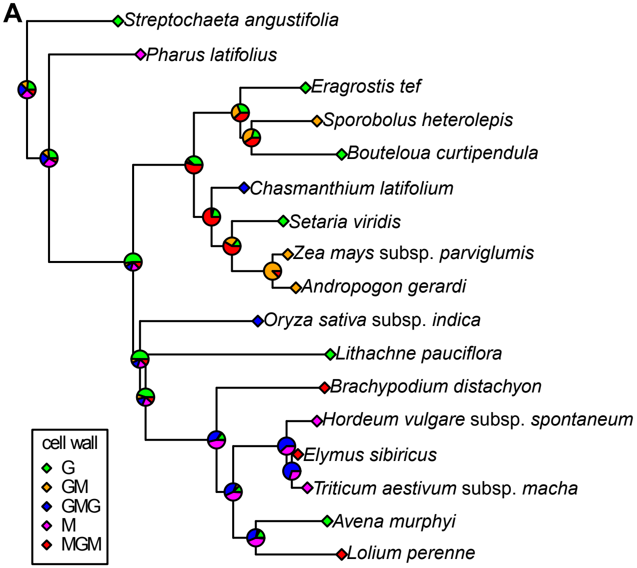

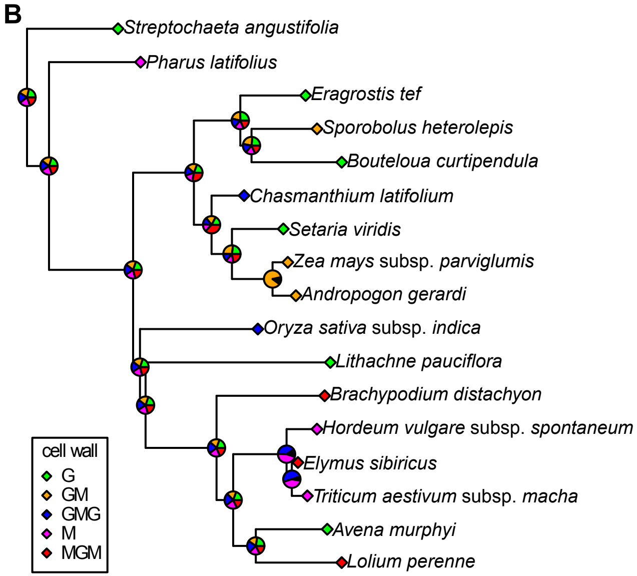

Appendix S5. Ancestral state reconstruction of cell wall composition. (A) All-rates-different model. (B) Equal-rates model. Green color: no lignin in AZ or surrounding cells (G). Orange color: nonlignification in the cells above the AZ and lignification in the cells below the AZ (GM). Blue color: lignified AZ cells sandwiched between nonlignified cells (GMG). Pink color: lignin in both the AZ and the surrounding cells (M). Red color: nonlignified AZ cells sandwiched between lignified cells (MGM).
